# Lactate metabolic reprogramming and histone lactylation modification in sepsis

**DOI:** 10.7150/ijbs.116088

**Published:** 2025-07-28

**Authors:** Ji Zhang, Dan Wu, Fu Zeng, Haiyun Gu, Chengbao Li, Juan P Cata, Kefang Guo, Changhong Miao, Hao Zhang

**Affiliations:** 1 Department of Anesthesiology, Zhongshan Hospital, Fudan University, Shanghai, China.; 2 Shanghai Key Laboratory of Perioperative Stress and Protection, Shanghai, China.; 3 Department of Anesthesiology, Shanghai Medical College, Fudan University, China.; 4 Department of Anesthesiology, Rizhao People's Hospital, Shandong, China.; 5 Department of Anesthesiology,Shandong Provincial Hospital affiliated to Shandong First Medical University, Jinan, Shandong, China.; 6 Department of Anesthesiology and Perioperative Medicine, The University of Texas-MD Anderson Cancer Center, Houston, TX, USA.; 7 Anesthesiology and Surgical Oncology Research Group, Houston, TX, USA.; 8 Department of Anesthesiology, Minhang Hospital Affiliated to Fudan University, Shanghai, China.

**Keywords:** sepsis, inflammation, lactylation, therapeutic targets

## Abstract

Sepsis, a serious condition characterized by life-threatening organ dysfunction owing to infection, lacks specific therapeutic interventions. Lactate serves as a crucial biomarker in sepsis, reflecting both the patient's metabolic state and the severity of the condition. Lactylation, the process whereby lactate is conjugated to lysine residues in proteins, profoundly alters protein structure and function. This review delves into the crucial roles of lactate and lactylation within the septic environment, illuminating the intricate feedback loop between metabolic reprogramming and lactylation in sepsis. Herein, fluctuations in lactate levels influence patterns of lactylation, which subsequently regulate energy metabolism. Lactylation is essential for modulating immune responses, adjusting gene expression profiles in immune cells, and shifting the balance between pro-inflammatory and anti-inflammatory pathways. The discovery of these pathways has significant implications for development of targeted therapies against sepsis. Furthermore, this review addresses the advancements and current limitations associated with lactylation research methodologies, and proposes new directions for future research. Overall, this narrative underscores the transformative potential of lactylation in understanding and managing sepsis, advocating for a multidisciplinary approach to unravel the complex interplay between metabolic processes and epigenetic regulation in critical illnesses.

## 1. Introduction

Sepsis arises from a dysregulated host response to infection. Severe sepsis and septic shock contribute to significant morbidity and mortality^1^, posing a substantial economic burden^2^. Despite substantial efforts to discover clinically useful diagnostic markers and develop effective medical management strategies, progress remains limited owing to the phenotypic heterogeneity of patients with sepsis^3^. Therefore, novel specific biomarkers and target approaches are urgently required.

Dysregulation of the host immune response is a major factor in the development of multiple organ dysfunction syndrome (MODS) and associated mortality in sepsis patients^4^. Sepsis-related deaths typically occur in susceptible hosts with a high microbial burden. In the initial phase of sepsis, an excessive pro-inflammatory response occurs^5^. This hyper-inflammatory state is driven by the immune system's overreaction to infection, leading to widespread activation of inflammatory pathways. In contrast, the later phase of sepsis is characterized by profound immune dysregulation. This state is marked by leukocyte and cytokine depletion, lymphocyte apoptosis, impaired antigen presentation, defective phagocytosis, and immune cell exhaustion^6^, ^7^. It heightens vulnerability to opportunistic infections, drives the development of multiorgan dysfunction, and is associated with increased fatalities^8^-^10^. Although the early and late stages of sepsis are distinct, they are interrelated. Throughout both phases, leukocytes undergo substantial metabolic alterations, generating metabolites such as adenosine, unsaturated fatty acids, and lactic acid, which may contribute to organ damage^11^.

Sepsis has long burdened human health and lacks specific diagnostic and therapeutic markers. Lactate, a byproduct of glucose metabolism, is a marker of sepsis severity^12^. The Sepsis-3 guidelines now recommend persistent serum lactate levels >2 mmol/L after adequate fluid resuscitation as a criterion for septic shock due to its strong association with disease severity and mortality^1^. In patients with sepsis, lactate accumulation is caused by increased lactate production and reduced clearance^13^.

Lactate controls immune responses, regulates energy metabolism, and serves as a signaling molecule regulating intercellular shuttling^14^. Zhao et al. identified histone lysine L-lactylation (K_L-la_) as an epigenetic modification, demonstrating that lactate directly facilitates K_L-la_^15^. In turn, lactylation modification regulates the level of lactate by acting on glycolytic pathway-related enzymes^15^. Beyond its metabolic role, lactate demonstrates significant pathophysiological relevance in sepsis pathogenesis by modulating immune cell via histone lactylation-dependent mechanisms, thereby reshaping systemic inflammatory responses^16^.

In this comprehensive review, we elucidate the intricate mechanisms that underlie lactylation, meticulously examine its profound impact on immune cells, and engage in a thoughtful discourse regarding its promising potential for both the diagnosis and treatment of sepsis.

## 2. Lactate Metabolism

### 2.1. Physiological lactate metabolism

Lactate was discovered by Karl Wilhelm Scheele in 1780, and in 1843, Scherer suggested its role in septic and hemorrhagic shock^17^. Subsequently, the paradoxical metabolic profile observed in malignant cells, characterized by 10-fold elevated lactate production under normoxic conditions (aerobic glycolysis), constitutes the biochemical hallmark of the Warburg effect. This metabolic rewiring, driven by tumor microenvironment acidosis and mitochondrial metabolic plasticity, establishes lactate not merely as waste but as a dynamic regulator of cellular energetics through redox shuttle mechanisms^13^, ^18^. Recent studies have indicated lactate's capacity to impose Warburg-like metabolic states in other fast-proliferating non-tumor cells^19^. For example, lactate influences inflammatory responses, memory formation, neuroprotection, wound healing, and ischemic injury^20^. In critically ill patients, elevated concentrations of serum lactate (hyperlactatemia) are related to a poorer clinical prognosis, and blood lactate kinetics may be used as a monitoring biomarker^21^.

**Figure 1:**
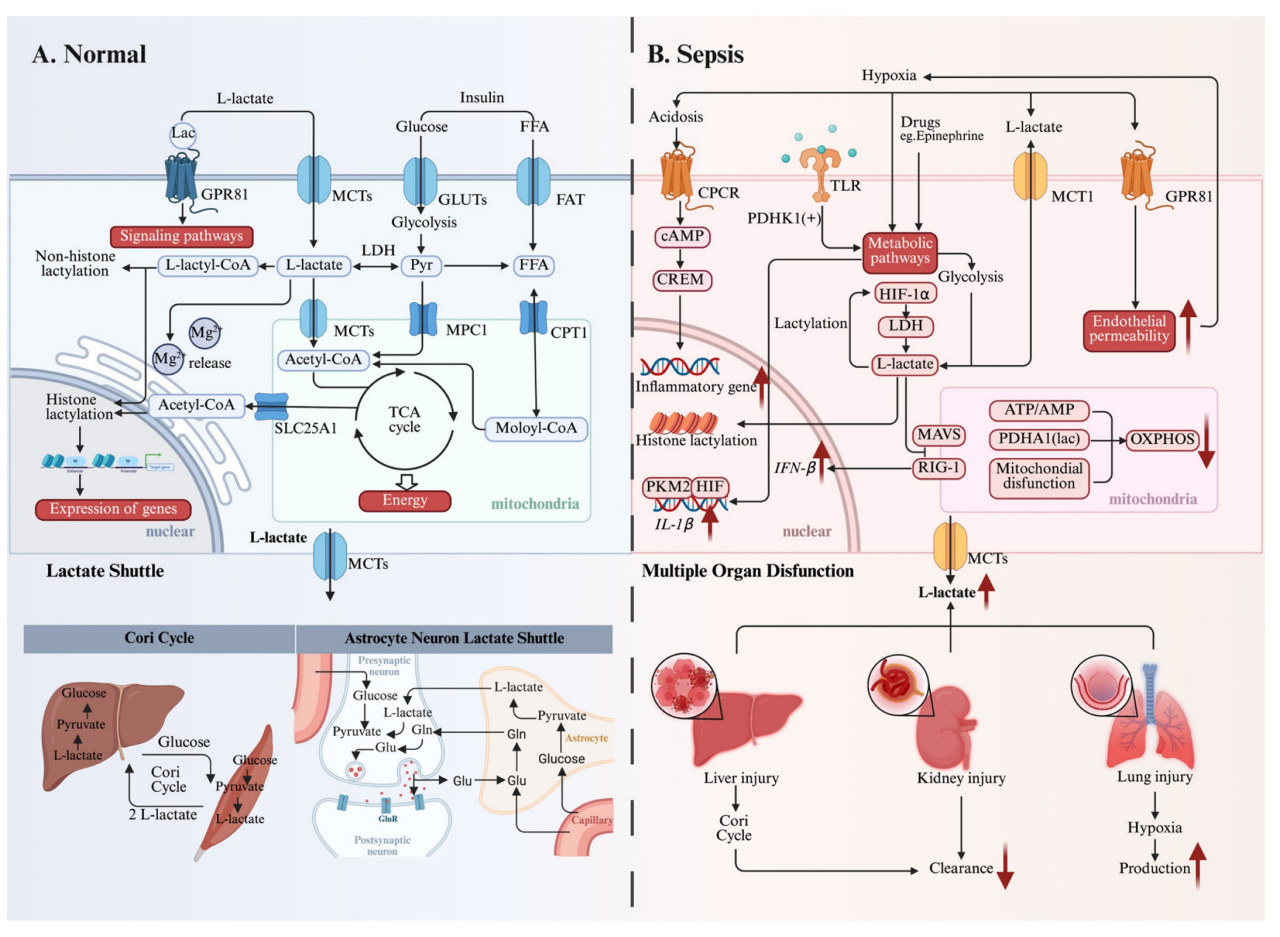
** Lactate metabolism changes under physiological and septic conditions.** Alterations in lactate metabolism occur in both physiological and pathological contexts, giving rise to diverse biological effects. (Left) Under physiological conditions, lactate serves as an important "fuel" that not only acts as a signaling molecule to regulate downstream pathways but also regulates gene expression and protein function through lactylation modifications. The lactate shuttle facilitates the flow of energy between cells and promotes intercellular communication, as seen in processes like the Cori Cycle and the astrocyte-neuron lactate shuttle. (Right) In septic conditions, factors such as hypoxia, drugs, and cytokines promote glycolysis and metabolic reprogramming, leading to increased lactate production, thereby exacerbating cellular damage. Furthermore, multiple organ dysfunction increases lactate production and reduces lactate clearance, further intensifying lactate acidosis. *Created with BioRender.com. **Abbreviations:** MCT: Monocarboxylate Transporter; GLUT: Glucose Transporter; FAT:; Fatty Acid Transporter; FFA: Free Fatty Acid; Glu: Glucose; Gln: Glutamine; CPCR: Cytokine Production and C-Reactive Protein; TLR: Toll-Like Receptor; CREM: cAMP Response Element Modulator; LDH: Lactate Dehydrogenase; OXPHOS: Oxidative Phosphorylation.

Lactate exists as two stereoisomers: L-lactate and D-lactate. L-lactate rotates polarized light clockwise (+), while D-lactate rotates it counterclockwise (-)^22^. At physiological pH, both L-lactic acid and D-lactic acid primarily exist as their conjugate bases, L-lactate and D-lactate, which have similar chemical and physical properties^23^. L-lactate, derived from glycolysis, is the primary lactate isomer in human metabolism. While trace amounts of D-lactate exist, mainly from gut microbiota or the methylglyoxal pathway, mammal cells overwhelmingly produce and utilize the L-enantiomer^20^, ^24^. Under oxygen-deprived conditions, the glycolytic pathway characteristically generates L-lactate as a terminal metabolic output **(Figure 1A)**. Under aerobic conditions, glucose undergoes catabolic processing through glycolysis, yielding pyruvate molecules and adenosine triphosphate (ATP) as immediate energy currency. The mitochondrial matrix subsequently imports pyruvate for enzymatic conversion to acetyl-CoA via pyruvate dehydrogenase complex (PDC) activity. This activated acetyl unit fuels the tricarboxylic acid cycle, driving electron transport chain-coupled oxidative phosphorylation to maximize ATP synthesis while liberating carbon dioxide as a metabolic byproduct. In contrast, oxygen deprivation triggers anaerobic metabolic reprogramming wherein lactate dehydrogenase (LDH) catalyzes pyruvate reduction to L-lactate. This enzymatic conversion, coordinately regulated by LDH and PDH activity gradients, maintains cellular redox homeostasis during compromised mitochondrial respiration^25^. Moreover, recent metabolomic studies have revealed a metabolic compartmentalization that glycolytic L-lactate acts as a systemic shuttle, bridging cytoplasmic glycolysis with mitochondrial TCA cycle activity across multiple organ systems^26^.

As a pivotal gluconeogenic precursor, L-lactate serves as a critical anaplerotic substrate by donating carbons to replenish hepatic glucose reservoirs. Mechanistically, L-lactate acts as a potent allosteric activator of hepatocyte gluconeogenic machinery, undergoing enzymatic reconstitution into glucose-6-phosphate via the Cori cycle. This newly synthesized glucose is subsequently exported into systemic circulation, establishing a metabolic relay system that sustains organismal energy homeostasis through iterative glucose-lactate interconversion^27^. According to the Randle Cycle theory, lactate acts as a key substrate in the glucose fatty acid cycle.

Beyond endogenous biosynthesis, cells acquire L-lactate and D-lactate through active import of extracellular pools via proton-coupled symport mechanisms. This transmembrane flux is mediated by evolutionarily conserved monocarboxylate transporters (MCTs/SLC16A), a family of pH-sensitive carrier proteins exhibiting tissue-specific isoform expression. The kinetic complementarity between low-Km MCT1 (ubiquitously expressed baseline transporter) and high-capacity MCT4 (stress-responsive efflux specialist) creates a dynamic lactate exchange network. Particularly, MCT1's nanomolar affinity enables precise regulation of monocarboxylate gradients, whereas MCT4's rapid turnover capacity (Km ≈ 25 mM) provides emergency clearance during glycolytic bursts, effectively preventing cytosolic proton accumulation. This fundamentally sustains the lactate shuttle mechanism, wherein oxidative cells abundantly expressing MCT1 actively uptake lactate generated by glycolytic cells predominantly expressing MCT4, thereby establishing an intercellular metabolic coupling that facilitates energy substrate redistribution^27^.

GPR81 belongs to a subfamily of G protein-coupled receptors, also known as hydroxycarboxylic acid receptors, that are known for their responsiveness to L-lactate. Distinct from canonical metabolic sensing mechanisms, this receptor transduces extracellular lactate signals via Gi/o-coupled cAMP attenuation without perturbing intracellular redox states^28^. Through this distributed signaling network, it orchestrates three-tiered physiological regulation, including reprogramming lactate-induced energy metabolism, preserving neuronal integrity, and regulating inflammation in adipose tissue, skeletal muscle, the central nervous system, and the cardiovascular system^28^. In addition, GPR81 affects blood glucose and lactate concentrations by influencing key biochemical pathways, including GLUT4 translocation and CPT1 activity, through modulation of insulin secretion and sensitivity^29^.

The identification of D-lactate in pathological conditions dates back to 1979^30^. For a considerable period, its presence in mammals was largely attributed to microbial activity within the gut^31^. However, recent investigations have challenged this exclusive view, demonstrating that mammalian cells can indeed produce D-lactate via glycolytic pathways^32^. This endogenous production often involves methylglyoxal (MGO), a highly reactive intermediate. MGO primarily arises as a side product during glucose catabolism, specifically from the non-enzymatic and side-enzymatic reactions of triose phosphates (dihydroxyacetone phosphate [DHAP] and glyceraldehyde 3-phosphate [G3P])^33^-^35^. Subsequently, the glyoxalase system catalyzes the conversion of MGO into D-lactate or glutathione (GSH)^36^. The glyoxalase system consists of two enzymes: glyoxalase 1 (GLO1) and glyoxalase 2 (GLO2)^37^, ^38^. GLO1, also known as S-D-lacoylglutathione lyase, is conserved across various species including humans, mice and yeast^39^. It facilitates the condensation of MGO with GSH, forming S-lactoylglutathione^40^. GLO2 then hydrolyzes this intermediate, yielding D-lactate and regenerating GSH^38^. However, given that physiological D-lactate levels are considerably lower than L-lactate, its precise physiological significance in humans remains an active area of research.

### 2.2 Lactate metabolism in sepsis

Inadequate oxygen delivery is a hallmark characteristic of sepsis, septic shock, and other types of shock. According to Suetrong and Walley^41^, inadequate oxygen delivery cannot fully explain the elevated lactate production in sepsis. They speculated that lactate concentrations may surpass normal levels even before true tissue hypoxia occurs, suggesting that other factors, such as enhanced glycolysis and drugs, may contribute to hyperlactemia. In addition, impaired tissue oxygen use and abnormal microcirculatory flow result in anaerobic metabolism, which causes pyruvate to be redirected towards lactate production**(Figure 1B)**^41^.

Under hypoxia, allosteric regulation of glycolysis occurs prior to hypoxia-inducible factor(HIF)-mediated transcriptional upregulation of glucose transporters and glycolytic enzymes^42^. HIF-1α boosts glycolytic flux by upregulating key enzymes (e.g., LDH-A) and glucose transporters, sustaining ATP production despite oxygen scarcity^43^, ^44^. Cells also reduce their reliance on oxygen by activating O_2_-dependent mitochondrial oxidative phosphorylation (OXPHOS) and prioritizing glycolysis to maintain sufficient ATP production. The ATP/AMP ratio is crucial for regulating enzyme activity via these mechanisms. Under hypoxic conditions, this ratio declines, leading to diminished allosteric inhibition by ATP on glycolytic enzymes such as phosphofructokinase and pyruvate kinase, thus enhancing flux through the glycolytic pathway. In addition, other mechanisms act as regulators of glycolysis under hypoxic conditions, including those dependent on the PI3K/Akt pathway^45^ and hypoxia-dependent post-translational modification (PTM) of glycolytic enzymes^46^.

Immune cells are central to the dysregulated inflammatory cascade in sepsis, contributing to elevated lactate levels. Khatib-Massalha et al.^47^ demonstrate that HIF-1α activation is essential for lactate generation in bone marrow neutrophils. The mobilization of neutrophils is facilitated by lactate through GPR81-mediated signaling in endothelial cells, which simultaneously decreases VE-cadherin expression and enhances vascular permeability in bone marrow. In activated T cells, TCR signaling upregulates pyruvate dehydrogenase kinase 1 (PDHK1) activity. This enzyme inhibits mitochondrial pyruvate utilization and subsequent promotion of lactate generation^48^. PDHK1 is also crucial for synthesis of cytokines, such as granzyme B, IFNγ, and TNFα^49^. Research by Palsson-McDermott et al.^50^ showed that the PKM2-HIF-1α complex exhibits direct binding capacity to the IL-1β promoter region, thereby facilitating metabolic reprogramming toward glycolysis and enhancing inflammatory activation. These findings suggest that in sepsis, lactate generation may primarily reflect inflammatory processes rather than merely functioning as a hypoxia biomarker.

Multiple clinical investigations have established a significant correlation between diminished lactate clearance capacity and unfavorable clinical prognosis^51^-^53^. In sepsis, mitochondrial dysfunction^54^ and PDH dysregulation^55^ impair OXPHOS, thereby exacerbating lactate accumulation within the cytoplasm. The body employs multiple compensatory mechanisms for lactate metabolism, including hepatic gluconeogenic conversion via the lactate shuttle system and renal excretion through urinary elimination. Notably, clinical observations demonstrate a positive association between serum lactate concentrations and severity scoring systems (SOFA/qSOFA)^12^, ^56^. This correlation indirectly reflects organ dysfunction because impaired organ function leads to reduced clearance of lactate^57^.

Significant concentrations of D-lactate are detectable across various pathological states. For instance, heightened D-lactate levels have been consistently observed within tumor microenvironments^58^, ^59^. Growing evidence now points to unique metabolic and signaling roles for D-lactate, particularly in contexts such as dysbiosis of the gut microbiota^60^, mitochondrial dysfunction, and certain metabolic derangements^61^. This expanding understanding suggests a potentially critical involvement of D-lactate in the progression of conditions like sepsis.

## 3. Lactate-Mediated Lactylation

### 3.1. Discovery of lactylation

**Figure 2:**
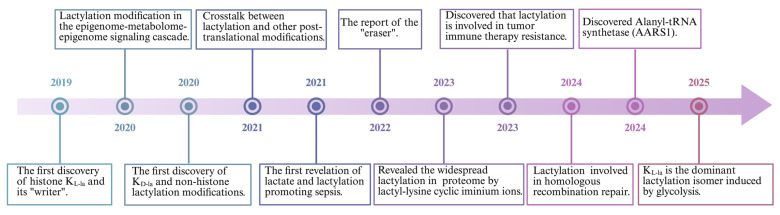
** A succinct historical overview of the development of lactylation.** Key discoveries in lactylation and its role in related diseases from 2019 to 2025. *Created with BioRender.com.

Beyond its well-established roles as an energy substrate and signaling molecule, lactate has been recently implicated as an epigenetic regulator through the discovery of protein lactylation **(Figure 2)**. This PTM exists in three isomeric forms: lysine lactylation (K_L-la_), N-ε-(carboxyethyl)-lysine (K_ce_), and D-lactoyl-lysine (K_D-la_). Each isomer originates from a distinct precursor: K_L-la_ from L-lactate, K_ce_ from MGO, and K_D-la_ from LGSH^24^. Notably, Kla is recognized as the predominant lactylation modification found on histones^24^.

Lactylation was initially reported by Zhang et al. in 2019^15^. Their foundational research established that L-lactate, a metabolic byproduct of glycolysis, functions as a direct precursor stimulating K_L-la_ on core histones. To validate this, they meticulously confirmed histone K_L-la_'s presence utilizing HPLC-MS/MS and immunoblotting with a pan-anti-Kla antibody. These investigations revealed a dose-dependent elevation of histone K_L-la_ levels in response to exogenous L-lactate stimulation. Furthermore, metabolic tracing experiments employing isotopic L-sodium lactate (^13^C_3_) and glucose (U-^13^C_6_) confirmed that endogenous lactate generation profoundly influences histone K_L-la_ abundance. Collectively, this seminal study introduced the concept of a "lactate clock", demonstrating that histone lactylation and acetylation exhibit distinct temporal dynamics in orchestrating gene expression to foster cellular homeostasis^15^.

The formation of K_D-la_ (D-lactoyl-lysine) and K_ce_ (N-ε-(carboxyethyl)-lysine) has been observed under conditions where the glyoxalase system is impaired. Gaffney and colleagues notably revealed that LGSH can act as an acyl donor for lactoyllysine formation^62^. They were the first to identify non-enzymatic K_D-la_ via chromatographic techniques and the alkyne-tagged methylglyoxal analogs. Their findings also indicated that this modification preferentially accumulates on glycolytic enzymes, implying a role in metabolic regulation^62^. Recently, Zhao et al. had revealed that nuclear factor-κB (NF-κB) signaling downregulates GLO2 via tristetraprolin-mediated mRNA decay in the glycolysis pathway^63^. This metabolic pathway leads to cytosolic accumulation of LGSH, which subsequently mediates post-translational protein modifications through D-lactyllysine formation. The reaction mechanism proceeds via a transient S-lactylated thiol intermediate (stabilized by proximal cysteine residues), followed by lactyl group transfer to neighboring lysine residues.

It's worth noting that Zhang and colleagues' research further distinguished the regulatory mechanisms governing K_L-la_ and K_D-la_. They found that K_L-la_ production is strictly dependent on changes in glycolytic flux but independent of glyoxalase enzyme expression. Conversely, K_D-la_ is influenced by the glyoxalase pathway but does not respond to glucose levels^24^. This distinction emphasizes the divergent metabolic origins of K_L-la_ and K_D-la_.

### 3.2. Selection of lactylation modification sites

#### 3.2.1. Histone lysine lactylation

Histones are a fundamental class of proteins located in the cell nucleus, which are responsible for tightly packaging DNA into the chromatin structure^64^. Chromatin organization is fundamentally maintained by nucleosomes, each consisting of an octameric histone core containing two copies each of H2A, H2B, H3, and H4^64^. These evolutionarily conserved proteins serve dual roles that provide structural integrity for DNA packaging and functioning as platforms for diverse post-translational modifications including methylation, acetylation, phosphorylation, and ubiquitination. Such epigenetic marks dynamically alter chromatin conformation, thereby modulating essential cellular functions ranging from transcriptional control to genome maintenance and repair mechanisms^65^.

The groundbreaking discovery of histone lysine lactylation as a novel PTM was initially achieved through proteomic analysis of both HeLa cells and murine bone marrow-derived macrophages^15^. Subsequently, lactylation modification sites were identified in other species including *Trypanosoma brucei*^66^, *Botrytis cinerea*^67^, and rice^68^. Moreover, the transcription factor, such as Glis1, induces H3 lysine 18 lactylation (H3K18la) via increasing the production of lactate, participating in the determination of the fate of pluripotent stem cell through the “epigenome-metabolome-epigenome” cross-cascade^69^. KL-la actively participates in these processes, whereas KD-la is generally undetectable on histones under physiological conditions^24^. Notably, a recent study demonstrated that inflammatory stimulation in GLO2-/- macrophages leads to the accumulation of D-lactyl-GSH, which in turn promotes a selective increase in histone lysine lactylation^70^. In vitro experiments suggest that this process may be mediated by the intermediate metabolite lactyl-CoA. Although D-lactyl-GSH is typically considered a precursor of KD-la modifications, the observed increases in lactylation were specific to L-lactylation at H3K79 and H3K18. Moreover, the data indicate potential interplay between lactylation and other PTMs. These observations raise important questions regarding the existence of K_D-la_ on histones, as well as the enzymatic or non-enzymatic mechanisms governing its deposition and its potential biological significance, all of which warrant further investigation.

Variations in identified histone lactylation sites likely arise from species-specific differences or spatiotemporal dynamics of lactylation. However, several key questions persist, including identifying specific chromosomal regions enriched with histone lactylation sites and clarifying the biological processes linked to these modifications. Extensive further investigation is crucial to unravel these aspects and fully grasp histone lactylation's significance.

#### 3.2.2. Lactylation of non-histone proteins

Lysine lactylation has also been observed in non-histone proteins. Comprehensive proteomic analyses have identified lactylated proteins across multiple subcellular compartments, spanning nuclear, cytoplasmic, mitochondrial, endoplasmic reticulum, and membrane-localized proteins, suggesting its broad regulatory potential in diverse biological processes^71^. The functional consequences and enzymatic regulation of these non-histone lactylation events appear to be determined by their specific subcellular distribution and protein-protein interaction networks. The functional consequences and enzymatic regulation of these non-histone lactylation events appear to be determined by their specific subcellular distribution and protein-protein interaction networks. A large number of non-histone Kla sites have been mapped, providing a foundation for understanding their biological significance^72^. Gaffney et al. reported the discovery of 350 proteins with D-lactic acid modifications, predominantly found in the glycolysis and carbon metabolism pathways^62^. In recent years, a plethora of cross-species discoveries have highlighted the ubiquity and diversity of lactic acid-mediated protein lactylation across multiple species^66^, ^68^. Furthermore, probing lactylation modifications has provided profound insight into how they influence the regulation of individual proteins.

High Mobility Group Box 1 (HMGB1), a ubiquitous nuclear protein, is well-established for its role in orchestrating inflammatory responses upon release from activated macrophages^73^. In the context of sepsis, macrophages take up extracellular lactate to instigate HMGB1 lactylation^63^. Further research highlights HMGB1's involvement in hypoxia/reoxygenation(H/R) conditions. Lactylational HMGB1 released from hepatocytes. This, in turn, promotes macrophage recruitment and inflammatory activation, ultimately contributing to the worsening of organ impairment^74^. The METTL family of methyltransferases catalyzes methylation modifications across diverse biomolecules including nucleic acids and proteins. Xiong et al. identified K281la and K345la of METTL3, which enhanced its binding to N^6^-methyladenine (m^6^A)-modified RNA and activated STAT3 signal transduction^75^. This regulatory mechanism encompasses both histone and non-histone lactylation events. In the tumor microenvironment, lactate-mediated upregulation of METTL3 enhances m^6^A-dependent signaling pathways, consequent immunosuppression and uproptosis^75^,^76^. These results suggest that lactylation exerts subtle regulatory effects at various sites.

### 3.3. Writers, erasers, and readers

**Figure 3:**
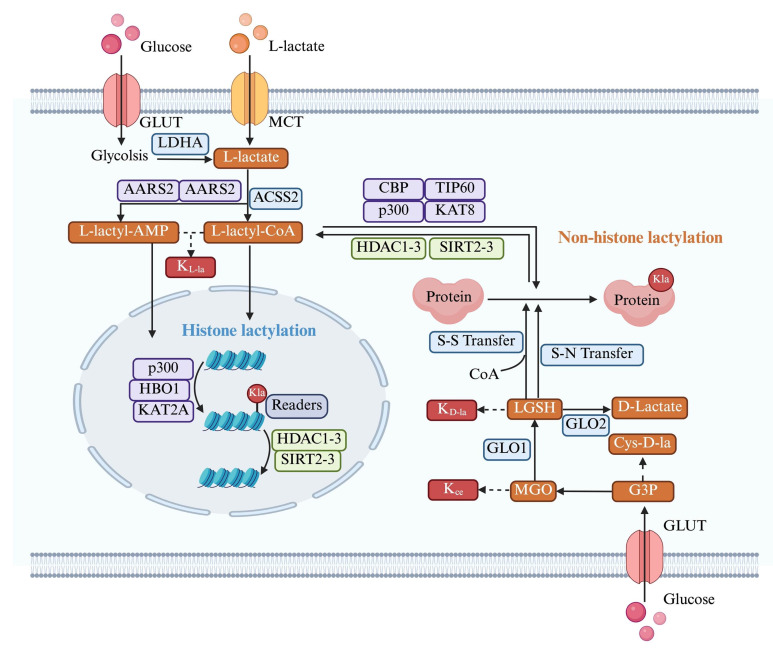
** Two different ways of lactylation generation.** Kla exists in two classic forms, including K_L-la_ and K_D-la_. L-lactate not only enters the nucleus to mediate histone lactylation but also modifies non-histone proteins through enzyme-catalyzed reactions. D-lactate modifies non-histone proteins through a non-enzymatic process. *Created with BioRender.com. **Abbreviation:** MCT: Monocarboxylate Transporter; LGSH: Lipoic Acid-Glutathione Conjugate; MGO: Methylglyoxal; GLUT: Glucose Transporter.

Lactylation is a reversible process involving covalent modification of proteins. Although the non-enzymatic lactylation process has been confirmed in the K_D-la_, most lactylation are dependent on enzymatic catalysis **(Figure 3)**. Parallel that of acetylation, L-lactate is typically converted into lactyl-CoA, which transfers to lysine residues by “writers” and is removed from lactylation modifications by “erasers”^77^. Evidence shows that lactylation shares these effectors with some other acylations^78^, such as malonylation, succinylation, glutarylation^79^ and 2-Hydroxyisobutyrylation^80^.

The L-lactate substrate for protein lactylation is acquired through two primary pathways: one is endogenous L-lactate generated from extracellular glucose via glycolysis; another is exogenous L-lactate directly transported into cells by MCTs. Therefore, the generation of L-lactyl-CoA by enzymes such as GTP-dependent succinyl-CoA synthetase (GTPSCS) or acyl-CoA synthetase short-chain family member 2 (ACSS2) is considered a catalytic prerequisite for this modification^81^, ^82^. One of the most classic histone acetyltransferases(HATs) is P300, which was first reported as a “writer” of lactylation in HEK293T cells^15^. Similarly, Cui et al. depleted P300 in bone marrow-derived macrophages (BMDMs) showing that K_L-la_ was considerably diminished^83^. The lactyltransferase activity of P300 and its homolog CREB-binding protein C (CBP), which mediate HMGB1 lactylation, is modulated through GPR81-β-arrestin2 signaling in macrophages^63^. Expanding our understanding of lactylation machinery, Chen et al. identified TIP60 as a lactyltransferase responsible for NBS1 K388 lactylation^84^, while Niu et al. characterized HBO1 as another key enzyme in this modification system^85^. Structural analysis revealed HBO1's catalytic E508 residue as essential for its activity on both histone, particularly H3K9la at transcription start sites, and non-histone substrates, underscoring its gene regulatory importance. Recent research indicates that L-lactate can be converted to lactyl-AMP, a process that is not entirely dependent on lactyl-CoA^86^, ^87^. The aminoacyl-tRNA synthetase family members AARS1/2 have emerged as evolutionarily conserved L-lactate sensors and lactyltransferases. They utilized ATP to directly append lactyl groups onto lysine residues, a process facilitated by the binding of L-lactate to the active site, E508. Li et al.^88^ established a genetic code expansion orthogonal system to study lactyl-lysine incorporation, demonstrating that AARS2-mediated cyclic GMP-AMP synthase (cGAS) lactylation at N-terminal sites disrupts its phase separation properties and impairs DNA sensing function, modulating stress response. Proteomic analyses have identified p53 as a key AARS1 target, with lactylation at K120 and K139 in its DNA-binding domain inhibiting phase separation, DNA binding, and transcriptional activity^87^. Notably, β-alanine competitively inhibits lactate-AARS1 interaction, suggesting therapeutic potential for slowing tumor progression. However, it remains uncertain whether AARS1/2 also function as "writers" of histone K_L-la_. Further investigation is required to validate and fully elucidate their potential role in this process^86^, ^88^.

LGSH serve as a lactyl group donor for protein lactylation, forming D-lactylation through a non-enzymatic reaction^89^. Research by Zang et al. has also uncovered a process where D-lactate from E. coli secretions stimulates K_D-la_ in HepG2 cells, potentially via a SCOT1-catalyzed biosynthesis of D-lactyl-CoA^90^. Furthermore, glyceraldehyde-3-phosphate (GAP) has been shown to induce lactylation modifications on proteins like Kelch-like ECH-associated protein 1 (KEAP1)^91^. Specifically, GAP-mediated D-lactylation of KEAP1 cysteine residues can alleviate its inhibitory effect on nuclear factor erythroid 2-related factor 2 (NRF2), leading to NRF2 activation^91^, ^92^ . However, the precise mechanism by which GAP induces cysteine lactylation remains to be fully elucidated.

In addition to “writers”, some “erasers” in relation to lactylation have been identified. Histone deacetylases (HDACs), enzymes involved in deactylation, are classified into four distinct classes (I-IV), differentiated by their cofactor dependencies. Classes I, II, and IV are Zn²⁺-dependent, while class III (sirtuins) requires NAD⁺^93^. Moreno-Yruela et al. reported that class I HDACs act as delactylases, demonstrating HDAC1-3 exhibit broad delactylase activity, targeting both K_L-la_ and K_D-la_ as well as other short-chain acyl modifications^94^. Despite various HDACs are expressed in the cytosol, the systematic identification of Kla erasers specifically targeting extranuclear proteins remains incomplete^95^. Evidence highlights sirtuins as key regulators of lactylation dynamics. Jennings et al. identified sirtuin 2 as a lactoyl-lysine eraser that removes lactyl groups from synthetic peptides associated with PKM2^96^. Furthermore, Zu et al. established SIRT2 as a multifunctional modulator of histone acetylation marks across diverse substrates, including synthetic peptides, nucleosomes, and cellular histones in neuroblastoma models^97^. Similarly, a SILAC-based quantitative proteomics and crystallographic study elucidated that the NAD+-dependent deacetylase SIRT3 facilitates the delactylation of non-histone proteins, CCNE2 K348la^98^. Research into the 'erasers' of K_D-la_ has identified HDAC1-3 as the most potent via in vitro experiment^62^. However, given their nuclear localization, HDAC1-3 likely target only a limited number of proteins found to exhibit K_D-la_ modifications^62^. Conversely, SIRT2 is more ubiquitously present in the cytoplasm and is thus considered a primary candidate for K_D-la_ cleavage^96^. Although studies on Kla effectors remain limited, existing research suggests that the stereospecificity of both 'writer' and 'eraser' enzymes will dictate the distinct kinetics and competitive effects observed between K_D-la_ and K_L-la_ modifications.

The characterization of Kla readers remains an important frontier for future research. Bromodomain-containing proteins, known to bind acylated histone marks and modulate transcriptional activity^99^, represent potential Kla readers. Notably, several histone acetyltransferases - including CREBBP, p300, PCAF, GCN5, and TAFII250 - have been identified as candidate interactors due to their conserved bromodomains and established roles in epigenetic regulation.

### 3.4. Glycolytic reprogramming and lactylation

The intricate interplay between glycolysis and protein lactylation in the context of metabolic reprogramming presents a compelling area of study. This complex interaction exerts crucial regulatory roles across a myriad of cellular pathways and physiological functions. One study emphasized the critical role of increased glycolytic activity in facilitating histone lactylation^15^. Glycolytic reprogramming indirectly modulates lactylation levels by enhancing lactate production because lactate is a crucial precursor for this modification. This connection results in a positive association between the glycolysis rate and the lactylation of lysine residues. Lactylation represents a critical PTM that transforms the structure and functionality of proteins involved in glycolysis, thereby affecting metabolic routes. Hypoxia is a key factor in glycolytic reprogramming, with specific mechanisms involving HIFα, c-Myc, and AMPK. For example, in pulmonary arterial hypertension, HIF-1α-mediated transcriptional activation of PDK1 and PDK2 drives lactate accumulation, which promotes pulmonary artery smooth muscle cell proliferation via H3K18la and H4K5la^100^. Furthermore, in human leukemic cells, HIFs regulate glycolysis independently of the control exerted by oncogenic pathways^100^. The oncoprotein c-Myc mirrors HIF-α in its capacity to upregulate glycolytic gene expression, thereby potentiating aerobic glycolysis. Chen et al. reported an NUSAP1-LDH-A-glycolysis-lactate feedforward loop^101^. NUSAP1 can bind to c-Myc and HIF-1α, not only directly enhancing glycolysis and lactate production in hepatocellular carcinoma (HCC) cells but also increasing NUSAP1 lactylation levels through a positive feedback loop, further promoting the proliferation of HCC cells. Moreover, hypoxia induces the accumulation of mitochondrial AARS2, which inactivates both enzymes by lactylating PDHA1 at lysine 336 and carnitine palmitoyltransferase 2 (CPT2) at lysines 457/8. These modifications functionally impair both enzymes, leading to suppressed oxidative phosphorylation through dual inhibition of acetyl-CoA generation from pyruvate and fatty acid oxidation.

Lactylation exerts multifaceted control over glycolytic pathways through both transcriptional regulation and structural modulation of key metabolic components. Li et al.^102^ uncovered a a self-amplifying circuit involving glycolytic flux, H3K18la modification, and the mitotic regulators TTK/BUB1B in PDAC. Their findings revealed that promoter-localized H3K18la enhances transcription of TTK and BUB1B, which in turn upregulates P300 expression to further potentiate glycolysis. Notably, TTK was shown to phosphorylate LDH-A at tyrosine 239, activating LDH-A enzymatic function and creating a reciprocal stimulation of both lactate production and H3K18la deposition. Parallel mechanisms have been observed in neuroinflammatory contexts. During Alzheimer's disease progression, activated microglia undergo metabolic reprogramming from oxidative phosphorylation to glycolysis, accompanied by accumulation of H4K12la at glycolytic gene promoters. This epigenetic modification drives transcriptional activation of glycolytic enzymes, establishing a pathological feedback loop involving H4K12la, PKM2, and sustained glycolytic activation that ultimately promotes microglial dysfunction and disease advancement^103^. Lactylation of fructose-bisphosphate aldolase A (ALDOA) at K147 significantly impairs its catalytic function, revealing an autoregulatory mechanism that enables cells to respond to excessive lactate accumulation and restore metabolic equilibrium^104^. Enrichment analysis has shown that lactylation particularly enriched among metabolic enzymes, with pronounced effects on core energy metabolism pathways, Biosynthetic processes and redox-sensitive enzymes^105^. Additionally, it notably affected oxidoreductases and dehydrogenases, demonstrating the substantial influence of lactylation on cellular metabolism and energy homeostasis^106^.

### 3.5. Crosstalk between lactylation and other post-translational modifications (PTMs)

**Figure 4:**
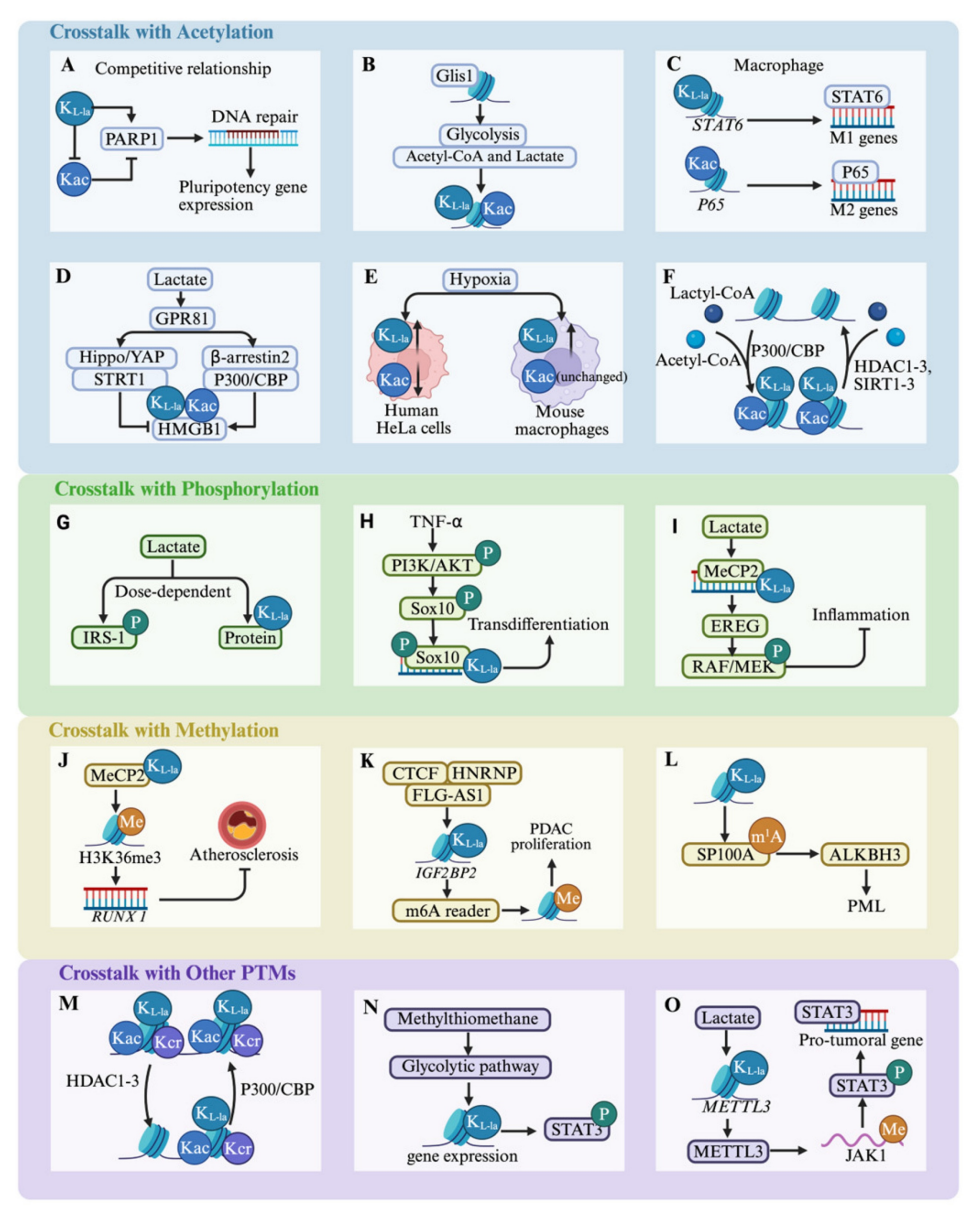
** Lactylation crosstalk with other post-translational modifications.** The majority of post-translational modifications (PTMs) do not exist independently. Two or more different PTMs can interact, and their combined states can influence each other. (A-F)There is a high degree of similarity and a close relationship between lactylation and acetylation, and their crosstalk is an important process linking metabolism and epigenetics. (G-I)Lactylation and phosphorylation together regulate the direction of cell growth and differentiation. (J-L)Lactylation and phosphorylation also play crucial roles in disease progression. (M-O)Numerous studies have explored the crosstalk between lactylation and other PTMs. *Created with BioRender.com. **Abbreviation:** K_L-la_: Lactylation; Kac: Acetylation; P: Phosphorylation; Me: Methylation; PDAC: Pancreatic Ductal Adenocarcinoma; PML: Promyelocytic Leukemia; PTM: Post-Translational Modification.

The functional activity of most proteins is mediated by their interactions with other proteins. A significant number of proteins undergo at least one PTM and frequently multiple PTMs, indicating that crosstalk among various PTMs on proteins is a common occurrence^107^ **(Figure 4)**. A deeper understanding of the crosstalk among PTMs could facilitate the exploration of lactylation modifications. Consequently, a focus on interrelated and regulated metabolic pathways is crucial to elucidate the connections between lactylation and other acylations.

A substantial degree of congruence and synchrony is observed between lactylation and acetylation; for instance, they share the same “effector”. P300 was identified the “writer” enzyme histone lysine lactyltransferase^15^, while certain HDACs were discovered as “erasers” with delactylase activity^94^. Given the shared regulatory machinery involving HATs and HDACs for acetylation and lactylation, respectively, these modifications likely exhibit functional interplay. Moreover, lactate serves as a common metabolic regulator of both processes. Lactate uptake triggers dual modifications of HMGB1 through distinct mechanisms: p300/CBP-dependent lactylation, and enhanced acetylation via SIRT1 inhibition through the Hippo/YAP pathway, coupled with β-arrestin2-mediated nuclear translocation of p300/CBP following GPR81 activation^63^. However, crosstalk between lysine acylations may occur because of their integration within cellular metabolic networks. The transcription factor Glis1 exemplifies this integration by differentially modulating chromatin architecture - decompacting glycolytic gene loci while condensing somatic gene regions to promote glycolytic flux. This metabolic shift elevates intracellular acetyl-CoA and lactate pools, which in turn increase H3K27ac and H3K18la at pluripotency-associated genomic loci, thereby establishing an open chromatin state conducive to cellular reprogramming^68^. In another study, researchers observed a distinct variation in the dynamic distribution of the histones, Kla and Kac, across mouse oocytes and early embryos^108^. Specifically, under hypoxic conditions, notable reductions were observed in both H3K23la and H3K18la within blastocysts, unlike consistent levels of H3K23ac and H3K18ac. These reductions have been implicated in the development of severe developmental impairments. Although the aforementioned evidence suggests a significant correlation between lactylation and acetylation, it is not reasonable to categorize their relationship as either synergistic or competitive. This is because variations in lactylation and acetylation differ markedly across different cells, including their occurrence, modification sites, and biological effects. Further research will provide a deeper understanding of PTMs.

In addition to lysine acetylation, a range of other acyl modifications such as crotonylation, succinylation, and butyrylation frequently coincide with lactylation on the same proteins, implying possible crosstalk between these PTMs. A comprehensive study integrating genome-wide profiling of H3K9ac, H3K9cr, and H3K18la dynamics with ATAC-seq and RNA-seq data revealed that histone crotonylation (Kcr) and Kla are widely distributed in brain tissues and exhibit strong correlations with chromatin accessibility and transcriptional activity^109^. HDACs can “erase” histones, Kcr and Kla. Moreover, in LPS-stimulated macrophages, PKM2 succinylation at K311 promotes its nuclear translocation, enhancing IL-1β and HIF-1α-dependent gene expression and driving aerobic glycolysis^78^. Conversely, SIRT5 serves as an “eraser” by effectively removing succinylation from PKM2 and activating it, which reverses lactate production^78^. Interestingly, these acylations, including crotonylation and 2-hydroxyisobutyrylation, also involve p300, a key “writer” protein that facilitates crosstalk^78^.

Substantial evidence suggests a close association between methylation and lactylation. Bioinformatic analyses show that H3K18la is predominantly found in active enhancers near functionally significant genes in the respective tissues. It also marks transcriptionally active CpG-rich promoters, including those of highly expressed housekeeping genes, across multiple tissue types^110^. Importantly, H3K18la exhibits a positive correlation with both H3K27ac and H3K4me3, as well as with overall transcriptional activity^110^. Furthermore, Chen et al.^111^ demonstrated that MeCP2 K271la, in conjunction with H3K36me3/RUNX1, contributes to the amelioration of atherosclerosis. The atheroprotective effects of exercise depend on the interaction between MeCP2 K271la and H3K36me3, which enhances chromatin accessibility and suppresses RUNX1 transcription. Therapeutic strategies enhancing MeCP2 lactylation promote the recruitment of reparative M2 macrophages, aiding plaque stabilization and reducing cardiovascular risk. Moreover, lactylation is associated with RNA methylation modifications that influence RNA processing, transport, translation, and stability, thereby indirectly regulating protein production and its functions within cells. A previous study revealed that deficiency in the m^4^C methyltransferase *METTL15* elevates oxidative stress, disrupts mitochondrial membrane potential, and alters metabolic flux, ultimately increasing lactate production and boosting H4K12la and H3K9la^112^. The regulation of methylation “effectors” can also promote disease progression via histone lactylation. Liu et al.^113^ demonstrated that the CTCF-HNRNPU axis, mediated by FLG-AS1, recruits EP300 to activate the m^6^A reader IGF2BP2, inducing lactylation at its promoter and accelerating pancreatic cancer growth^113^. Conversely, lactylation upregulates ALKBH3 while disrupting tumor-suppressive promyelocytic leukemia protein (PML) nuclear bodies via m^1^A demethylation of SP100A, promoting cancer aggressiveness^114^. In conclusion, the dynamic crosstalk between proteins and RNA PTMs represents a largely unexplored frontier, warranting a comprehensive investigation to uncover its regulatory complexity and biological significance.

## 4. Lactylation in Inflammation

### 4.1. Macrophages

Tissue-resident and newly recruited macrophages differentiate into two functionally distinct subsets: the classically activated (M1) and alternatively activated (M2) phenotypes. M1 macrophages, typically induced by pathogen-associated molecular patterns (e.g., LPS) or Th1 cytokines (e.g., IFN-γ and GM-CSF), exhibit a pro-inflammatory profile characterized by enhanced production of inflammatory mediators^115^. Conversely, M2 polarization occurs in response to Th2 cytokines (e.g., IL-4, IL-13, and IL-10), resulting in an anti-inflammatory phenotype that contributes to tissue repair and immunoregulation^116^.

Kelly et al.^117^ showed that activation by pro-inflammatory stimuli leads macrophages to shift their metabolism towards glycolysis and reduces their reliance on OXPHOS, akin to the Warburg effect seen in tumors. Key metabolic byproducts, such as lactate, accumulate in the cytoplasm and influence various aspects of macrophage behavior. A growing body of evidence indicates that lactate mediates the lactylation of histone and non-histone proteins, thereby regulating macrophage phenotypes. M1-polarized macrophages exposed to bacteria were used as a model to demonstrate that lactate in these cells could be transformed into lactyl-CoA. This compound then enters the nucleus, initiating an intrinsic “lactate clock”^15^. As a result, genes characteristic of the M2 phenotype that are modified by lactylation are activated, leading the cells to exhibit M2-like attributes. In late-stage M1 polarization, increased histone lactylation upregulates homeostatic genes (e.g., Arg1) involved in tissue repair. Wang et al.^118^ established that this modification enhances the anti-inflammatory and pro-angiogenic potential of macrophages through reparative gene activation, particularly improving cardiac recovery post-myocardial infarction via IL-1β-mediated GCN5 recruitment to H3K18la sites. Further studies have indicated that lactylation at H4K12 can also influence cellular inflammation, alongside H3K18la. Furthermore, in the investigation into the mechanisms underlying diabetic cardiomyopathy, Ma et al.^119^ observed that environments rich in free fatty acids preferentially utilize H4K12la and H3K18la to drive HIF-1α stabilization and the production of pro-inflammatory cytokines, such as IL-1β. In the context of neurodegenerative diseases, exemplified by Alzheimer's disease, which is pathologically characterized by chronic neuroinflammation and the progressive accumulation of misfolded proteins, lactylation is emerging as a critical modulator of both macrophage effector functions and the overall trajectory of neuroinflammation. Pan et al.^103^ demonstrated that H4K12la activates a glycolysis-H4K12la-PKM2 feedback loop by increasing LDH-A, PKM2, and HIF-1α expression, exacerbating neuroinflammation in Alzheimer's disease microglia. Lactylation also mediates the regulation of tumor progression and immune evasion within tumor microenvironments. The rapid proliferation of tumor cells leads to the production of lactate, which can induce histone lactylation in tumor-associated macrophages (TAMs), thereby influencing genes associated with the anti-tumor immune response. In glioblastoma, this modification promotes IL-10 expression through tumor-derived factor signaling while suppressing the PERK-ATF4 pathway, thereby leading to monocyte-derived macrophages toward an immunosuppressive phenotype^120^. Dysregulation of the GLO2/SLG/D-lactylation axis contributes to inflammatory disorders. Pharmacological targeting of GLO2, which results in the inhibition of lysine D-lactylation (K_D-la_), has been demonstrated to attenuate immune hyperactivation and mitigate inflammatory pathology in both in vitro and in vivo models^89^. Evidence suggests that PKM2 K62L-la can effectively suppress the Warburg effect, thereby facilitating a phenotypic switch in macrophages from a pro-inflammatory to a reparative state^121^. This discovery underscores the multifaceted regulatory roles of lactylation in metabolic processes. In summary, lactylation serves as a critical nexus, linking metabolite fluctuations, arising from various pathological conditions, to subsequent metabolic reprogramming and epigenetic modulation within macrophages. Consequently, targeting the lactylation pathway represents a promising therapeutic avenue for a spectrum of inflammation-associated diseases, including myocardial inflammation, neuroinflammation, oncogenesis, sepsis, and various forms of immune dysfunction.

**Figure 5:**
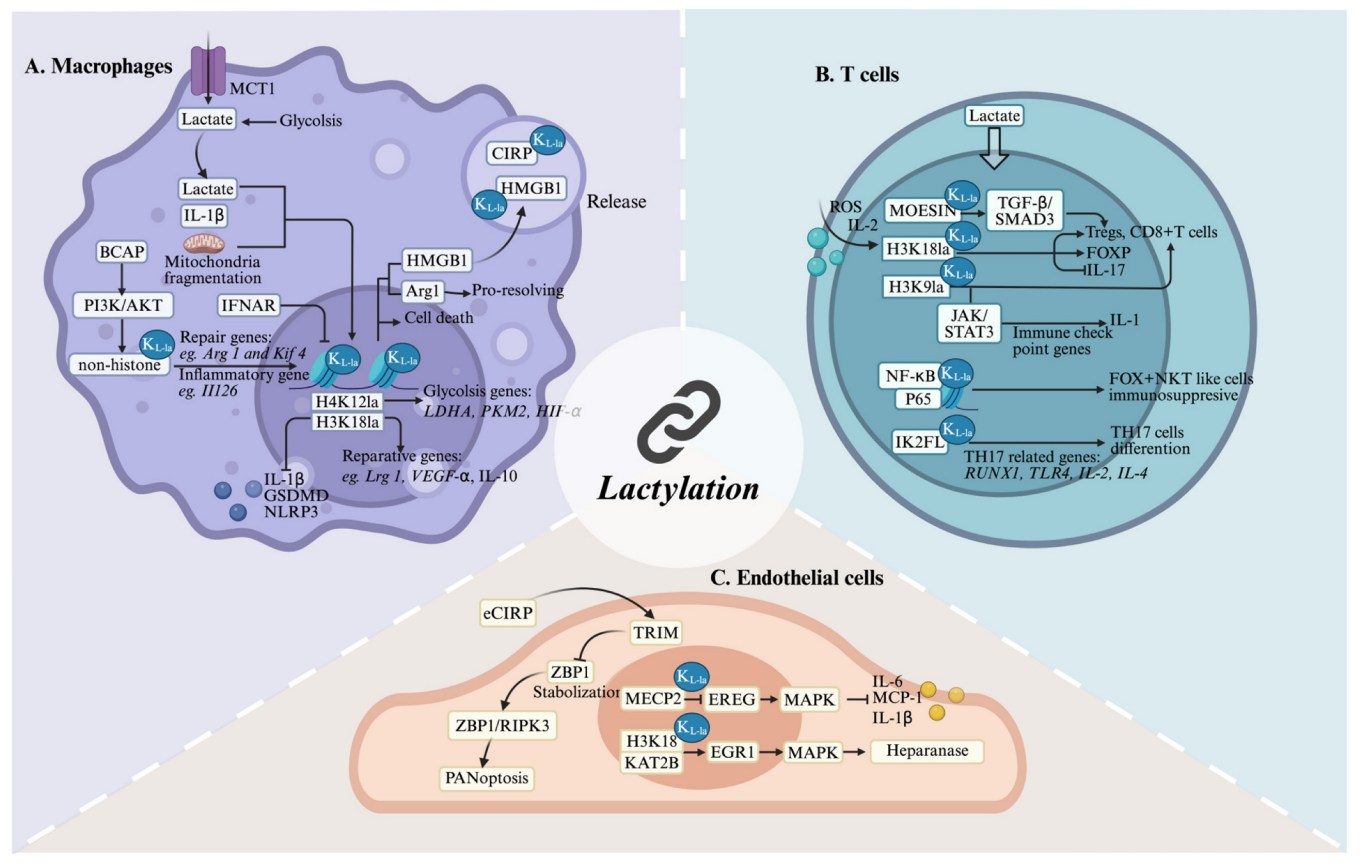
** Lactylation effects on inflammation-related cells.** (A) In macrophages, factors such as lactate, IL-1β, and mitochondrial fragmentation promote lactylation, whereas IFNAR inhibits histone lactylation. Lactylation regulates the expression of genes related to immunity, energy metabolism, and tissue repair. Additionally, lactylated proteins are released via vesicles into other cells, facilitating intercellular communication. (B) In T cells, lactylation regulates T cell differentiation and cytokine expression. (C) In endothelial cells, lactylation is involved in regulating panoptosis and cytokine expression. *Created with BioRender.com. **Abbreviation:** K_L-la_: Lactylation.

Emerging evidence highlights the crucial role of mitochondrial dynamics and epigenetic regulation in macrophage functional plasticity. Susser et al. reported that mitochondrial fragmentation is essential for pro-resolving macrophage responses, directly mediating post-inflammatory epigenetic reprogramming through histone lactylation^122^. Complementing these findings, Irizarry-Caro et al. showed that TLR and its adapter, BCAP, are critical cell-intrinsic switches in the shift from inflammatory to reparative macrophages through epigenetic alterations^123^. In addition, TRIM33, which contains a bromodomain, has been highlighted as a new reader of histone lactylation that potentially facilitates the connection between Kla and macrophage polarization. In addition to endogenous lactate, macrophages actively import extracellular lactate via MCTs, fueling lactylation of HMGB1. This modified HMGB1 is subsequently secreted via exosomes, exacerbating endothelial barrier dysfunction in polymicrobial sepsis^63^. Collectively, these studies demonstrate the pleiotropic effects of site-specific histone lactylation **(Figure 5A)**, revealing a sophisticated epigenetic network that orchestrates macrophage polarization and function in various pathological contexts.

### 4.2. T cells

Lactylation modulates T cells immune function by influencing the activity of intracellular transcription factors and regulatory proteins. T lymphocytes constitute the central mediators of adaptive immunity, comprising functionally distinct subsets including CD4⁺ and CD8⁺ T cells, natural killer T (NKT) cells, and γδ T cells^124^. CD4⁺ T cells differentiate into helper (Th) and regulatory (Treg) subsets that orchestrate immune activation and tolerance, respectively. Recent studies have highlighted the critical role of lactate-induced histone lactylation in modulating T cell function and immune responses. In Th17 cells, exogenous lactate leads to elevated global H3K18la levels. This, in turn, suppresses IL-17 secretion while upregulating Foxp3 expression through ROS-dependent IL-2 production^125^. Furthermore, higher lactate concentrations mitigate the pathogenic potential of Th17 cells. Naïve CD4+ and CD8+ T cells undergo sequential stages of activation, clonal expansion, and differentiation, with lactylation emerging as a key regulatory mechanism. In autoimmune uveitis, lactate-mediated lactylation was shown to govern CD4+ T cell differentiation. Specifically, Ikzf1 hyperlactylation at Lys164 promotes TH17 polarization by upregulating TH17-associated genes scuh as Runx1, Tlr4, IL-2, and IL-4, whereas delactylation at this site disrupts TH17 development^126^. These findings collectively highlight the therapeutic potential of lactate in intestinal inflammation. Furthermore, lactate is recognized as an essential component for enhancing the stability and functionality of Treg cells through lactylation. In the tumor microenvironment, lactate-mediated lactylation of MOESIN at Lys72 facilitates TGF-β receptor I binding and activates SMAD3 signaling, thereby promoting Treg cell generation^127^. Moreover, H3K18la modulates NF-κB p65 transcriptional activity, and elevated TNFR2 expression on Tregs correlates with unfavorable clinical outcomes in malignant pleural effusion^128^. CD8+ T cells (cytotoxic T lymphocytes), secrete cytotoxic substances that directly destroy target cells. The activation and effector function differentiation of CD8+ T cells are also regulated by lactylation. Research indicates that H3K18 and H3K9 lactylation are enriched in human CD8+ T cells, where they act as transcriptional enhancers^129^. Metabolic heterogeneity across CD8+ T cell subsets is associated with specific patterns of H3K18 and H3K9 lactylation, which are intricately linked to antitumor immunity. Intriguingly, intratumoral bacteria, such as Escherichia coli, can influence T cell behavior via lactylation. For example, lactate producted by E. coli- enhances retinoic acid-inducible gene 1 lactylation, inhibiting NF-κB binding to the Nlrp3 promoter in macrophages. This suppression reduces NLRP3 levels, subsequently amplifying Treg immunosuppression and CD8+ T cell antitumor responses^130^. Lactate that is transported into macrophages activates transcription by inducing H3K18la, thereby enhancing pro-tumor macrophage activity. However, M2 macrophages induce an immunosuppressive tumor microenvironment in HCC by inhibiting CD8+ T cell enrichment and reducing the proportion of interferon-γ positive CD8+ T cells^131^. Genome-wide CUT&Tag profiling demonstrated that H3K9 lactylation upregulates IL-11 expression through the JAK2/STAT3 signaling pathway in CD8+ T cells, contributing to immune checkpoint activation and functional exhaustion, which are associated with diminished immunotherapy responses in patients^132^. Natural killer T (NKT) cells, which exhibit hybrid NK and T cell features, include type 1 NKT, type 2 NKT, and NKT-like subsets. These cells secrete cytokines such as IFN-γ, IL-4, and IL-17, playing a multifaceted role in immune responses^124^. Single-cell RNA sequencing (scRNA-seq) analysis identified that FOXP3+ NKT-like cells express MCT1 and LDH-B, enabling lactate uptake and utilization to sustain their immunosuppressive function and hyperlactylated state in malignant pleural effusion^133^.

In conclusion, T cells are pivotal for mediating cellular immune responses and regulatory mechanisms in inflammatory environments** (Figure 5B)**. However, investigations into the role of lactylation in these processes are preliminary. Therefore, more comprehensive and detailed studies are needed in this area.

### 4.3 Endothelial cells (ECs)

Endothelial cells (ECs), which constitute the inner vascular lining, directly involve in the orchestration of inflammatory responses. These cells are not merely passive barriers; they also actively recruit immune cells, present antigens, and regulate hemostasis. Increasing evidence suggests that lactylation within ECs is involved in immune responses and closely associated with disease prognosis **(Figure 5C)**. In a murine atherosclerosis model, exercise training was found to elevate Mecp2 K271la in aortic ECs. This modification suppresses epiregulin (Ereg) expression by promoting Mecp2 binding to its chromatin. Consequently, reduced Ereg levels modulate MAPK signaling, downregulating pro-inflammatory mediators including Mcp-1, IL-1β, and IL-6, thereby attenuating atherogenesis^134^. Furthermore, lactate induces lactylation in pulmonary ECs and degrades the glycocalyx, subsequently worsening survival outcomes in patients with sepsis-induced acute lung injury (S-ALI). The lactyltransferase KAT2B enhances H3K18la deposition at the EGR1 promoter, increasing EGR1 transcription and upregulating heparanase, which exacerbates ALI^135^. Interestingly, lactylation of cold-inducible RNA-binding protein (CIRP) in macrophages induces the release of CIRP during sepsis. Extracellular CIRP (eCIRP) reduces the proteasomal degradation of ZBP1 by competitively binding to ZBP1 and blocking its interaction with TRIM32 after internalization by pulmonary vascular ECs (PVECs). This stabilization of ZBP1 enhances the ZBP1-receptor-interacting protein kinase 3 (RIPK3)-dependent PANoptosis in PVECs, thereby exacerbating the severity of post-sepsis ALI^136^. While these findings highlight the dual roles of EC lactylation in inflammation, further studies are needed to delineate lactylation-dependent signaling networks across different inflammatory phases.

## 5. Lactylation is a Biomarker and Therapeutic Target in Sepsis

Clinically, dynamic changes in circulating lactate levels not only serve as diagnostic markers but also correlate strongly with disease severity and patient outcomes^137^, ^138^. While lactate measurement remains a cornerstone of sepsis management, its utility as a standalone biomarker is limited by moderate clinical specificity. The recommendation strength for lactate is classified as “weak”, and current research suggests that more intricate regulatory mechanisms are involved. The characterization of histone lactylation represents a paradigm shift in lactate biology, revealing its direct role in epigenetic regulation. This breakthrough has opened new research directions for investigating lactate's multifaceted functions across various disease states, particularly in infection and sepsis. A deeper mechanistic understanding of lactylation mediated pathways may yield novel diagnostic approaches and therapeutic strategies for sepsis management.

In the initial phase of sepsis, immune cell activation drives lactate generation through aerobic glycolysis. Chu et al.^139^ identified H3K18la could serve as a dual-purpose biomarker for diagnosing and predicting in septic shock. The extent of H3K18la correlates with infection severity and may influence macrophage polarization by suppressing pro-inflammatory cytokines while enhancing anti-inflammatory responses. Interestingly, Yang et al.^63^ showed that suppressing lactate synthesis or blocking GPR81-dependent signaling markedly lowers exosomal HMGB1 concentrations in circulation, thereby enhancing survival rates in polymicrobial sepsis. These findings support the potential of targeting lactate and its associated signaling networks as a therapeutic approach for sepsis. Heat shock protein A12A (HSPA12A), an atypical HSP70 family member, uniquely modulates lactate metabolism. HSPA12A overexpression results in a reduction in glycolysis-dependent lactate production, thereby attenuating histone lactylation and HMGB1 release in hepatocytes. This mechanism impedes macrophage chemotaxis and subsequent inflammatory activation^74^. Histone lactylation is an important regulatory mechanism in inflammatory responses. Qiao et al.^140^ showed that histone lactylation triggers the RhoA/ROCK/Ezrin cascade, which promotes NF-κB activation, increases apoptosis and aggravates renal impairment in sepsis. Their work identified Ezrin K263 as the predominant lactylation site, underscoring the role of lactylation in metabolic reprogramming and inflammatory responses within renal tubular epithelial cells during sepsis-associated acute kidney injury (S-AKI).

Numerous preclinical studies have shown that inhibition of lactylation sites or associated signaling pathways may alleviate sepsis pathogenesis and improve patient outcomes **(Table S1)**. Through bioinformatics analysis and other techniques, Li et al.^141^ revealed significant correlations between sepsis and lactylation-associated genes such as S100A11 and CCNA2, suggesting their potential utility as diagnostic biomarkers and therapeutic targets. For drug resistant sepsis cases, Ma et al. identified methylsulfonylmethane (MSM) as an effective immunomodulatory agent against methicillin-resistant Staphylococcus aureus (MRSA) infections^142^. Their research showed that MSM promotes M2 macrophage polarization through the lactate-H3K18la-Arg1 axis, revealing a novel mechanism for combating antimicrobial resistance in sepsis management. Polymorphonuclear neutrophils (PMNs), key immune cells in the defense against bacterial infections, were investigated by Zhu et al.^143^, who highlighted the role of lactate-driven HMGB1 lactylation in triggering AKI in mice via the HMGB1-NETs signaling pathway. Their findings revealed that pretreatment with either glycyrrhizin (an HMGB1 inhibitor) or oxamate (an LDH-A inhibitor) effectively suppressed lactate/LPS-induced neutrophil extracellular trap (NET) formation. This intervention significantly reduced circulating and PMN-derived NET levels, ultimately mitigating AKI associated with lactate accumulation.

Mitochondrial fission triggers a series of detrimental events including mitochondrial dysfunction, energy depletion, amplified inflammation, and organ damage^144^. An et al.^145^ identified that lactate induces lactylation of mitochondrial fission 1 protein (Fis1) at lysine 20 (Fis1 K20la), which promotes mitochondrial fission, resulting in ATP depletion, excessive mitochondrial ROS production, and apoptosis. Notably, PDHA1 hyperacetylation and subsequent inactivation further amplify lactate accumulation, creating a vicious cycle that worsens S-AKI. Importantly, interventions to lower lactate levels or block Fis1 lactylation mitigated S-AKI, highlighting potential therapeutic avenues. In parallel, Lu et al.^135^ identified KAT2B as a lactyltransferase that catalyzes EGR1 lactylation via its GNAT domain during S-ALI. In vivo studies revealed that either lactate reduction or EGR1 knockout attenuated glycocalyx degradation and ALI severity while improving survival rates in murine sepsis models. These findings collectively position lactylation as a promising biomarker and therapeutic target in sepsis. However, compared to its well-explored role in cancer, lactylation-focused sepsis therapies remain understudied. Further investigation into lactylation mechanisms could yield critical insights for developing precision treatments for septic patients.

## 6. Methods in Lactylation Research

**Figure 6:**
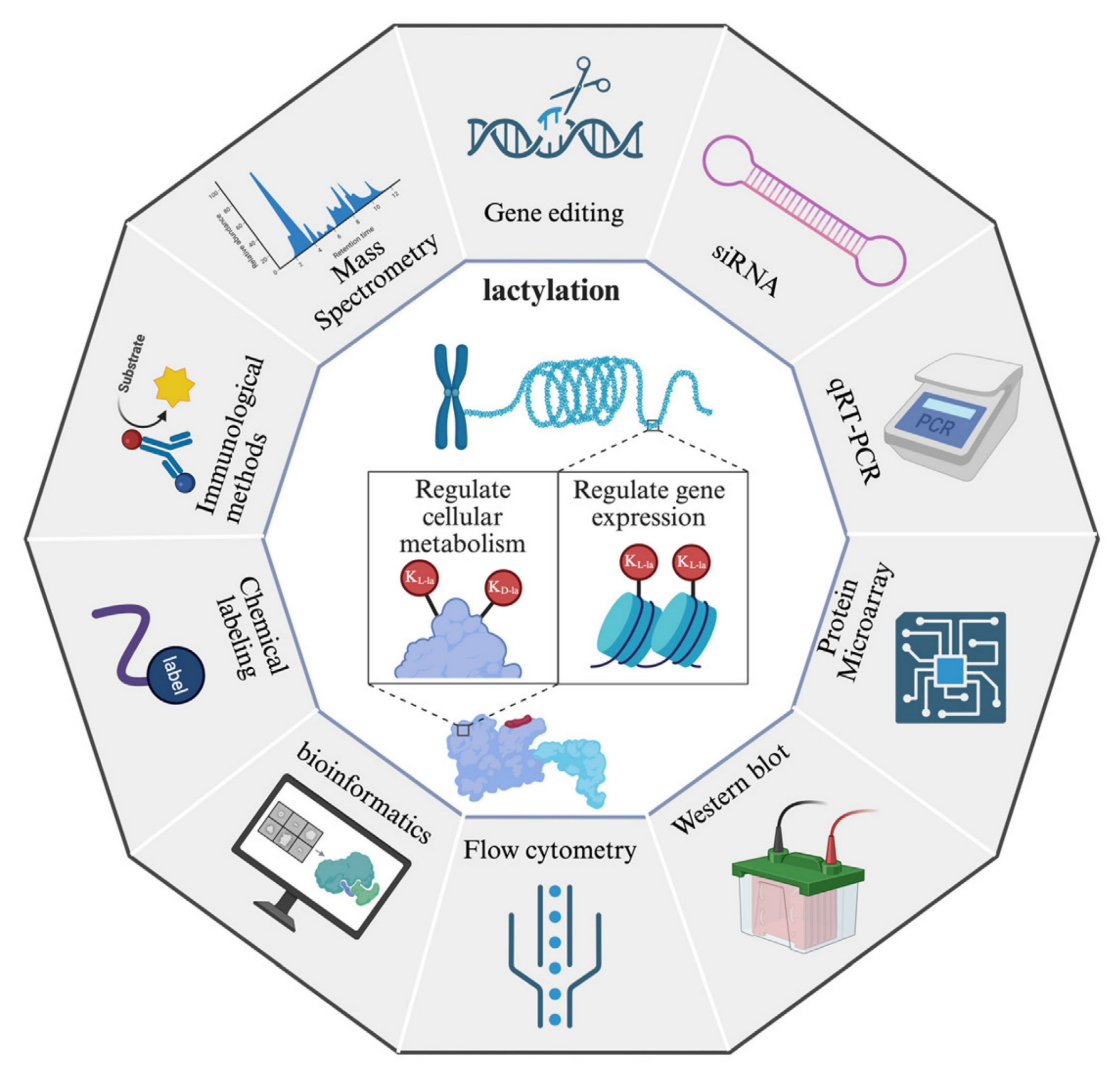
** Methods in lactylation research.** The diagram presents various techniques employed for the detection, characterization, and functional analysis of lactylation. Gene editing and siRNA-mediated knockdown are used to investigate the regulatory mechanisms of lactylation. qRT-PCR and protein microarrays enable the analysis of gene and protein expression changes associated with lactylation. Western blot and immunological methods, including antibody-based detection, facilitate the identification of lactylated proteins. Mass spectrometry provides high-resolution characterization of lactylation sites and quantitative analysis. Flow cytometry allows single-cell analysis of lactylation-related changes, while bioinformatics tools support data interpretation and pathway analysis. Additionally, chemical labeling techniques enhance the detection and enrichment of lactylated proteins. Together, these methodologies provide a comprehensive toolkit for studying lactylation and its role in cellular processes. *Created with BioRender.com.

The investigation of protein lactylation has progressed alongside advancements in detection and analytical methodologies, providing more precise and comprehensive insights into its biological functions **(Figure 6)**.

In the realm of proteomics, specific antibodies targeting lactylated peptides and bioorthogonal chemical probes designed for metabolic labeling represent indispensable tools for comprehensive investigation. Early seminal work by Zhang et al. leveraged a pan anti-KL-la antibody in conjunction with high-performance liquid chromatography-tandem mass spectrometry (HPLC-MS/MS) to initially identify 28 KL-la modification sites on core histones in both human and mouse cells^15^. A significant methodological advancement occurred in 2024 with the development of their second-generation antibody, which exhibits enhanced specificity, enabling the effective discrimination among K_L-la_, K_D-la_, and K_ce_ modifications^24^. This improved antibody significantly refines the resolution and accuracy of lactylation modification analyses. Bioorthogonal chemical analogs have been firmly established as a robust and versatile platform for both the metabolic labeling and subsequent proteomic characterization of diverse PTMs^146^. Complementing this, Sun et al. recently introduced YnLac, an alkynyl-functionalized bioorthogonal chemical reporter, as a novel methodology for the detection of lactylated proteins^147^. All in all, these methodological innovations provide a high-performance chemical framework that is critical for advancing lactylation research, thereby facilitating the elucidation of expanded networks of modified proteins and uncovering novel functional dimensions of this pervasive modification. Another major breakthrough came with Wan et al.'s discovery of a cyclic immonium ion derived from lactyllysine during tandem mass spectrometry, which served as a diagnostic marker for confident Kla site assignment^104^. By combining this signature ion with affinity-enriched lactylproteomics and large-scale spectral library comparisons, they substantially expanded the known lactylome, revealing widespread lactylation across non-histone proteins in both enriched samples and native proteomes.

Complementing experimental approaches, computational methods have emerged as efficient tools for Kla prediction. Jiang et al.^148^ addressed key bioinformatics challenges by constructing the first benchmark dataset for Kla and developing a few-shot learning framework capable of handling limited and imbalanced training data while avoiding overfitting. This newly designed predictor, FSL-Kla, serves as both an advanced tool for Kla site profiling and a generator of candidate sites for further experimental validation. In parallel, Lv et al.^149^ introduced DeepKla, the first computational framework specifically designed for Kla prediction in plants, which demonstrated exceptional accuracy through its innovative architecture combining supervised embedding layers, convolutional neural networks, bidirectional gated recurrent units, and attention mechanisms. Similarly, Lai et al.'s Auto-Kla employed automated machine learning (AutoML) to enable rapid and precise Kla site identification in gastric cancer cells^150^. Combining machine learning with experimental approaches enhances the validation of predicted site accuracy. For instance, one study successfully established a hypoxia-lactylation gene signature for PDAC that effectively predicted clinical outcomes and immunotherapy responses by combining multiple machine learning algorithms with single-cell analysis^151^. Notably, research on Kla sites has primarily focused on elucidating associations with specific biological pathways using advanced analytical techniques such as mass spectrometry or antibody arrays. This method mirrors the panel sequencing used in disease research for the targeted detection of specific gene mutations, offering a cost-effective alternative to comprehensive Kla proteome analysis. Nonetheless, this approach has inherent scientific limitations, including the potential exclusion of Kla sites not represented in the panel and heavy reliance on preexisting biological knowledge, factors that can significantly constrain the depth and breadth of the analysis and consequent discoveries. To effectively address these challenges and advance the field, enhancing existing methodologies using more comprehensive datasets is essential to significantly improve the accuracy and scope of biomarker discovery across various pathological conditions^93^. Distinguishing Kla from Kac is essential to discern their unique contributions to cellular mechanisms. Employing molecular techniques such as immunoblotting and mass spectrometry allows for the simultaneous analysis of these modifications on identical residues, which is critical for delineating the specific roles of Kac. The use of lysine (K) to glutamine (Q) substitution (K-to-Q) models of Kac's effects provides insights into its biological effects. The experimental induction of Kla using lactate, along with the co-stimulation of Kla and Kac with glucose, requires a meticulous experimental design to ensure that the specific effects of each modification are distinctly understood^93^. However, the methods used to mimic Kla are limited. Traditional point mutations that remove Kla do not replicate its biochemical environment as effectively as the K-to-Q substitution for Kac, highlighting a gap in the methods available for studying Kla. Although point mutations in lysine facilitate the removal of Kla, there is no direct analog of the K-to-Q substitution that effectively simulates Kac. Gene codon expansion technology offers a sophisticated means of artificially integrating Kla into target proteins. Nevertheless, this approach necessitates the coordinated expression of three heterologous components: engineered suppressor tRNAs, orthogonal aminoacyl-tRNA synthetases (aaRS), and synthetic lysine analogs in eukaryotic hosts, which creates substantial technical barriers for widespread implementation^152^. Building on these methodologies, Shao et al.^153^ developed an integrated workflow that combines proteomics with genetic code expansion, coupled with a unified functional assessment system. This approach not only facilitates precise detection of protein lactylation markers but also enables *in vivo* biosynthesis of position-specific lactylated proteins and systematic evaluation of their physiological roles. Such an integrative protocol provides unprecedented insights into the mechanistic aspects of post-translational regulation and its influence on cellular processes, creating a valuable paradigm for subsequent investigations into protein modifications. Furthermore, research should address the crosstalk between various PTM types. The possible interplay between lysine lactylation and other modifications, which may form a complex signaling network influencing septic processes, warrants deeper investigation.

*In vivo* studies utilizing animal models remain indispensable for deciphering the pathophysiological mechanisms and functional implications of protein lactylation in sepsis. Several animal studies have relied primarily on LPS-induced models that rapidly trigger systemic inflammation by activating a singular inflammatory pathway. Although these models offer a convenient experimental platform, they fail to recapitulate the multifaceted nature of human sepsis, particularly the complexity of polymicrobial infection and the nuanced regulation of the inflammatory response^154^. To address these constraints, the cecal ligation and puncture (CLP) model has emerged as a more clinically relevant alternative. By incorporating polymicrobial infections along with a gradually evolving inflammatory cascade, the CLP model more accurately mirrors the pathophysiological state of clinical sepsis, thereby enabling studies on the dynamic regulation of lactate production and clearance within a more clinically relevant context^155^. Furthermore, gene-edited mouse models (e.g., YAP^flox^/TAZ^flox^ and Lyz2-Cre mice^63^) have been used in sepsis research. These models enable a more refined investigation of the mechanisms underlying immunometabolic regulation, cellular energy homeostasis, and organ failure. Although animal models provide a robust mechanism for simulating the pathophysiological processes of sepsis, the complexity of *in vivo* environments and physiological discrepancies between humans and other species necessitate the use of *in vitro* cell line models for etiological research and clinical translation. Current research employs various murine cell lines to elucidate the immune responses within the septic milieu. These include the murine RAW 264.7 macrophage line^63^, murine PMNs^143^, mouse alveolar macrophages^136^, and induced bone marrow-derived macrophages^156^, each serving to highlight the different facets of immune activation and response in sepsis. Additionally, mouse pulmonary microvascular ECs^135^ and MLE12 cells^157^ have been used to explore the effects of sepsis on different organ systems. Additionally, human-derived cell lines are integral to this research area, with the human embryonic kidney cell line, HEK293T, of particular interest for its high transfection efficiency, making it a valuable tool for genetic studies and manipulation. The HK-2 cell line, which originates from the proximal tubule epithelia of adult kidneys, is essential for examining the specific impact of lactylation on cellular functions. These human cell lines are critical for understanding how acetylation influences cellular energy dynamics and inflammatory responses, thereby providing a controlled environment that facilitates detailed mechanistic studies.

## 7. Conclusions and Perspectives

Sepsis is a life-threatening systemic inflammatory syndrome typically associated with infection, and marked by profound metabolic alterations in immune cells, particularly the aberrant activation of the glycolytic pathway under aerobic conditions. Although lactate is a byproduct of glycolysis, its use as a marker of sepsis severity or prognosis remains controversial. Lactate fluctuations are closely related to sepsis and are among the most important characteristics worthy of further clinical evaluation. Beyond its metabolic role, lactate also functions as a signaling molecule, driving histone lactylation and engaging in crosstalk with other epigenetic modifications to directly influence transcriptional regulation^158^. A complex feedback network exists between metabolic reprogramming and lactylation in sepsis, finely regulating the inflammatory response and highlighting the adaptability of chromatin dynamics to metabolic changes. Lactate production pathways and their roles in lactylation are promising targets for therapeutic interventions. Modulating these pathways may offer new strategies for managing sepsis by altering both metabolic and epigenetic states.

Although significant progress has been made in characterizing protein lactylation, several critical questions remain unanswered in this emerging field. The current understanding of this post-translational modification presents numerous challenges that warrant further investigation. First, existing studies have primarily identified lysine residues as lactylation sites, leaving open the question of whether other amino acid residues may also undergo this modification. This knowledge gap highlights the need for more comprehensive proteomic analyses. Second, it remains unclear as to whether Kla has specific readers. Furthermore, in cases where lactylation occurs in conjunction with other PTMs, it remains unclear how different enzymes may influence the magnitude or specificity of Kla modifications. Third, the potential competition between lactylation and other acylation modifications remains unclear. Given the dynamic interactions and possible crosstalk between Kla and other acylation marks, such as the ε-amino group of lysine, which can undergo acetylation, methylation, or ubiquitination^159^, it remains unclear as to which experimental approaches can disentangle the specific effects of Kla. Fourth, Emerging evidence suggests Kla exhibits dynamic changes across disease states and cellular conditions. In sepsis particularly, the stage-specific functional consequences of lactylation on both histone and non-histone proteins demand systematic characterization. Fifth, the interaction between Kla and other acylation modifications generate a complex array of effects. It remains to be ascertained just how the potential off-target effects of this intricate network of epigenetic modifications can be mitigated. Sixth, Sun et al.^76^ demonstrated that Cu²⁺, rather than lactate itself, acts as the upstream activator of METTL16-mediated lactylation. Given the complexity of intracellular signaling networks, there may be previously unrecognized pathways that link copper ions to lactate metabolism. Moreover, it remains unclear as to whether lactylation induction is contingent on the presence of lactate, or requires a specific threshold concentration. Seventh, several lactylation-targeting compounds are currently undergoing clinical trials and show promising results in cancer therapy^160^. In light of preclinical data from sepsis studies, clinical indications for these drugs should be defined considering the phenotypic heterogeneity of patients with sepsis. Finally, while current investigations have predominantly centered on histone lactylation, the identification and functional characterization of this modification in non-histone proteins, such as Fis1^145^, represents a critical yet understudied area of research. Given the pivotal biological roles of nonhistone proteins, a more comprehensive examination of lactylation sites in both histones and nonhistones is imperative to advance our understanding of this intricate modification.

The emergence of artificial intelligence offers transformative potential for bridging these knowledge gaps. Recent breakthroughs in deep learning-based protein folding prediction have revolutionized structural biology^161^, ^162^. For proteins and PTMs with established structural data, computational simulations, in conjunction with databases like the Protein Data Bank^163^, facilitate the exploration of how conformational dynamics influence both the structural integrity of the protein and its interactions within cellular networks^164^. If structural data are unavailable, *in situ* simulations utilizing advanced algorithms, such as AlphaFold, offer valuable insights into the potential regulatory enzymes responsible for Kla modification^165^. By simulating Kla-modified proteins or peptides, molecular docking studies can be performed to assess the binding affinities of candidate enzymes, thereby refining the list of potential regulatory molecules. These computational predictions can be subsequently validated through experimental approaches in the laboratory. This integrative strategy, which combines cutting-edge computational tools with well-established biochemical principles, holds promise in providing a more nuanced and mechanistic understanding of how Kla regulates protein function and cellular processes.

Collectively, while investigations into lactylation in sepsis remain at a preliminary phase, accumulating evidence underscores its pivotal role as a molecular bridge connecting metabolic reprogramming and immunological regulation. Kla has been established as a central modulator of multiple sepsis-related pathological cascades, including but not limited to inflammatory signaling, angiogenic processes, metabolic derangements, and fibrotic remodeling. Mechanistically, the lactate-Kla axis orchestrates complex pathophysiological adaptations that drive disease progression through these interconnected pathways. Of particular clinical significance, lactylation exerts profound and multifaceted influences on both innate and adaptive immune responses during sepsis pathogenesis. These findings position lactylation as: (1) a promising diagnostic biomarker with potentially superior specificity compared to conventional lactate measurements, and (2) a novel therapeutic target for precision medicine approaches in sepsis management. Future research should focus on elucidating the spatiotemporal dynamics of lactylation in different sepsis stages and subtypes, which may ultimately establish this modification as a more reliable molecular indicator than lactate alone for clinical decision-making in critical care settings.

## Supplementary Material

Supplementary table.
