# Supplementary material for: Lactate metabolic reprogramming and histone lactylation modification in sepsis
Source: Int J Biol Sci. 2025 Jul 28;21(11):5034–55. doi: 10.7150/ijbs.116088 (PMC12374839; doi:10.7150/ijbs.116088)
Supplement: Supplementary file 1 — Supplementary table. [file ijbsv21p5034s1.pdf]

Table 1. Preclinical Investigations of Targeted Therapeutic Strategies in Sepsis

Targets	Mechanism	Results	Animal models and cell lines	Diseases	References
2-DG	HK2 inhibitor	Lung tissue pathological injury, accumulation of neutrophil, oxidative stress, expression of proinflammatory factors.	Mouse model, Primary murine peritoneal macrophages	ALI	[1]
2-DG	HK2 inhibitor	Protective effects against sepsis-induced AKI through the promotion of autophagy via the lactate/SIRT3/AMPK signaling pathway.	Mouse model, HK-2 cells	Sepsis-induced AKI	[2]
Lonidamine	HK2 inhibitor	Mitigates inflammatory damage; lonidamine directly interacts with the inflammasome ligand ASC, thereby preventing its oligomerization.	Mouse model, BMDMs	Autoimmune disease-multiple sclerosis (MS), ischemic stroke and sepsis	[3]
Sodium oxamate	LDHA inhibitor	Reduces circulating exosomal HMGB1	YAP^{flox}/TAZ^{flox}, Lyz2-Cre , and wild-	Sepsis	[4]

		levels and enhances survival outcomes in polymicrobial sepsis.	type mice; Murine RAW 264.7 macrophages		
Celastrol	PKM2 inhibitor	Binds Cys424 of PKM2 to inhibit aerobic glycolysis, Cys106 of HMGB1 to reduce IL-1β secretion, and Cys residues in LDHA.	Mouse; RAW264.7 cells	Sepsis	[5]
4-OI	GAPDH inhibitor	Downregulates aerobic glycolysis in activated macrophages, leading to anti-inflammatory effects.	Mouse; RAW 264.7 cells	Endotoxaemia	[6]
Sodium oxamate, 2-DG, STM2457,	LDHA inhibitor, HK2 inhibitor, METTL3 inhibitor,	Inhibits septic hyper-lactate-induced ferroptosis in alveolar epithelial cells and alleviates lung injury.	Mouse;MLE12 cells	Sepsis-associated lung injury	[7]
Sodium oxamate	LDH inhibitor	Alleviated glycocalyx degradation and ALI, improving survival outcomes in mice with polymicrobial sepsis.	Mouse; primary murine pulmonary microvascular endothelial cells	Sepsis-induced ALI	[8]
Glycyrrhizin;	HMGB1	Reversed the	Mouse	Lactate-	[9]

oxamate	inhibitors; LDH inhibitor	lactate- and low-dose LPS-induced upregulation of NETs in both blood and PMN cell supernatants, thereby alleviating AKI associated with lactate accumulation..	model, HK-2 cells	induced AKI	
GSK2837808 A	LDH inhibitor	Reduced lactate production, attenuated the 3-TYP-mediated increase in Fis1 K20la, and mitigated AKI.	Mouse model, HK-2 cells	Sepsis-induced AKI	[10]
BAY-876	GLUT1 inhibitor	Alleviated inflammation and apoptosis	Mouse model; HK-2, TCMK-1 and HEK293T cell	Sepsis-associated AKI	[11]

Abbreviation: 2-DG: 2-deoxyglucose; HK2: Hexokinase 2; ASC: Apoptosis-associated speck-like protein containing a CARD; AKI:Acute kidney injury; ALI:Acute lung injury; BMDMs: Bone marrow-derived macrophages; GLUT1: Glucose Transporter 1; LDHA: Lactate dehydrogenase A; LDH: Lactate dehydrogenase; TCMK-1: Tubular Cell line derived from Mouse Kidney – 1; HEK293T: Human Embryonic Kidney 293 Transformed.

1. Zhong WJ, Yang HH, Guan XX, Xiong JB, Sun CC, Zhang CY, et al. Inhibition of glycolysis alleviates lipopolysaccharide-induced acute lung injury in a mouse model. J

Cell Physiol. 2019; 234: 4641-54.

2. Tan C, Gu J, Li T, Chen H, Liu K, Liu M, et al. Inhibition of aerobic glycolysis alleviates sepsis-induced acute kidney injury by promoting lactate/Sirtuin 3/AMPK-regulated autophagy. Int J Mol Med. 2021; 47.

3. Chen C, Zhou Y, Ning X, Li S, Xue D, Wei C, et al. Directly targeting ASC by lonidamine alleviates inflammasome-driven diseases. J Neuroinflammation. 2022; 19: 315.

4. Yang K, Fan M, Wang X, Xu J, Wang Y, Tu F, et al. Lactate promotes macrophage HMGB1 lactylation, acetylation, and exosomal release in polymicrobial sepsis. Cell Death Differ. 2022; 29: 133-46.

5. Luo P, Zhang Q, Zhong TY, Chen JY, Zhang JZ, Tian Y, et al. Celastrol mitigates inflammation in sepsis by inhibiting the PKM2-dependent Warburg effect. Mil Med Res. 2022; 9: 22.

6. Liao ST, Han C, Xu DQ, Fu XW, Wang JS, Kong LY. 4-Octyl itaconate inhibits aerobic glycolysis by targeting GAPDH to exert anti-inflammatory effects. Nat Commun. 2019; 10: 5091.

7. Wu D, Spencer CB, Ortoga L, Zhang H, Miao C. Histone lactylation-regulated METTL3 promotes ferroptosis via m6A-modification on ACSL4 in sepsis-associated lung injury. Redox Biol. 2024; 74: 103194.

8. Lu Z, Fang P, Li S, Xia D, Zhang J, Wu X, et al. Lactylation of Histone H3k18 and Egr1 Promotes Endothelial Glycocalyx Degradation in Sepsis-Induced Acute Lung Injury. Adv Sci (Weinh). 2025; 12: e2407064.

9. Zhu L, Zheng Q, Liu X, Ding H, Ma M, Bao J, et al. HMGB1 lactylation drives neutrophil extracellular trap formation in lactate-induced acute kidney injury. Front Immunol. 2024; 15: 1475543.

10. An S, Yao Y, Hu H, Wu J, Li J, Li L, et al. PDHA1 hyperacetylation-mediated lactate overproduction promotes sepsis-induced acute kidney injury via Fis1 lactylation. Cell Death Dis. 2023; 14: 457.

11. Qiao J, Tan Y, Liu H, Yang B, Zhang Q, Liu Q, et al. Histone H3K18 and Ezrin Lactylation Promote Renal Dysfunction in Sepsis-Associated Acute Kidney Injury. Adv Sci (Weinh). 2024; 11: e2307216.
